# Syphilis among pregnant women in Northeast Brazil from 2008 to 2015: a trend analysis according to sociodemographic and clinical characteristics

**DOI:** 10.1590/0037-8682-0199-2019

**Published:** 2020-03-16

**Authors:** Kathleen Cézar de Mélo, Aisla Graciele Galdino dos Santos, Alyne Barbosa Brito, Saulo Henrique Salgueiro de Aquino, Érika Tenório dos Santos Alencar, Elena Maria da Silva Duarte, Michael Ferreira Machado, Maria Deysiane Porto de Araújo, João Paulo Silva de Paiva, Rodrigo Feliciano do Carmo, Thiago Cavalcanti Leal, Adeilton Gonçalves da Silva, Leonardo Feitosa da Silva, Divanise Suruagy Correira, Victor Santana Santos, Carlos Dornels Freire de Souza

**Affiliations:** 1 Universidade Federal de Alagoas, Departamento de Medicina, Arapiraca, AL, Brasil.; 2 Universidade Federal de Alagoas, Núcleo de Estudos em Medicina Social e Preventiva, Arapiraca, AL, Brasil.; 3 Universidade Federal do Vale do São Francisco, Colegiado de Ciências Farmacêuticas, Petrolina, PE, Brasil.; 4 Universidade Federal de Alagoas, Faculdade de Medicina, Maceió, AL, Brasil.; 5 Universidade Federal de Alagoas, Programa de Pós-graduação em Saúde da Família, Maceió, AL, Brasil.; 6 Universidade Federal de Alagoas, Núcleo de Epidemiologia e Saúde Pública, Arapiraca, AL, Brasil.

**Keywords:** Syphilis, Pregnant Women, Ecological Studies, Epidemiology

## Abstract

**INTRODUCTION:**

The number of syphilis cases among pregnant women in Brazil has increased. This study aimed to analyze the temporal trend of syphilis indicators among pregnant women in Northeast Brazil.

**METHODS:**

A time-series study was performed.

**RESULTS:**

We observed an increase in the detection rate of syphilis among pregnant women, those aged 15-19 years, and those of brown ethnicity. A strong correlation was observed between the detection rate of syphilis and family health strategy coverage.

**CONCLUSIONS:**

Despite an increase in primary care coverage, The increase in cases of syphilis among pregnant women is still considered a challenge.

About 1 million individuals are diagnosed with sexually transmitted infections (STIs) daily worldwide, thereby indicating that more than 364 million cases are reported annually. Nearly 357 million cases are attributed to four diseases, namely, trichomoniasis (143 million), chlamydia (131 million), gonorrhea (78 million), and syphilis (5.6 million)^1^.

Syphilis, or lues (lues venerea, Latin for venereal plague), is a systemic STI with chronic evolution caused by *Treponema pallidum*, a pathogen that exclusively infects humans and is an obligate intracellular parasite^2^. Although sexual transmission is the most common form of transmission, mother-to-fetus transmission during the gestational period has been an important public health issue due to the increased risk of premature birth, mortality, low birth weight, and impaired childhood development^1^ ^-^ ^3^. 

Approximately 1 million pregnant women are infected by syphilis annually worldwide, resulting in more than 300,000 fetal and neonatal deaths and exposure in more than 200,000 children who are at risk of premature death^4^. In Latin America and the Caribbean, approximately 166,000-344,000 babies with congenital syphilis are born annually^4^. In Brazil, a higher number of syphilis cases among pregnant women were observed in recent years. From 2015 to 2018, 119,903 syphilis cases among pregnant women were reported, of which 22.7% were in the Northeast Region^5^.

Due to the severity of this problem, the World Health Organization launched The Global Elimination of Congenital Syphilis: Rationale and Strategy for Action in 2007 based on four pillars, which were as follows: 1) ensuring sustained political commitment and advocacy; 2) increasing the quality of maternal and child health services; 3) identifying and treating pregnant women and their partners; and 4) establishing surveillance, monitoring, and evaluation systems^6^. In accordance with this global strategy, Brazil launched a protocol for the prevention of mother-to-fetus transmission of HIV and syphilis, with the goal of diagnosing and treating pregnant women with these diseases^7^. 

As syphilis has been an indicator of the quality of prenatal care, the monitoring of syphilis trends in this population is required to assess the progress of the interventions adopted^6^ ^,^ ^7^.

Thus, this study aimed to analyze the trend of gestational syphilis indicators from 2008 to 2015 according to sociodemographic and clinical characteristics in Northeast Brazil, which is composed of nine states. The registrations made after 2015 are considered preliminary and may still change. Thus, they were not included in the study. 

For this purpose, we performed an ecological time-series study. Six indicators of syphilis were selected, which were as follows: 1) detection rate per 1,000 live births (LB) among pregnant women with syphilis per year of diagnosis, 2) proportion of syphilis cases among pregnant women according to age group, 3) proportion of syphilis cases among pregnant women according to ethnicity, 4) proportion of syphilis cases among pregnant women according to formal education, 5) proportion of syphilis cases among pregnant women according to gestational age, and 6) proportion of syphilis cases among pregnant women according to clinical classification. In addition, we obtained the annual coverage of Brazil’s Family Health Strategy (FHS) during the study period.

Data were obtained from the Secretariat of Health Surveillance database (http://indicadoressifilis.aids.gov.br/) and the Brazilian Ministry of Health’s Department of Primary Care (http://dab.saude.gov.br/).

A trend analysis was performed using the joinpoint regression model, which can help classify indicator trend (stable, increasing, or decreasing) and annual percentage change (APC). The cross-correlation function (CCF) was used to describe the relationship between the rate of syphilis and FHS coverage. We considered a 95% confidence interval (95% CI) and a significance level of 5%. Analyses were performed using the Joinpoint Regression Program version 4.5.0.1 (National Cancer Institute, the USA) and gretl version 2019d (GNU General Public License).

This study did not require approval from the ethics committee as it utilized open public domain data without the identification of individuals.

Between 2008 and 2015, a total of 30,314 cases of syphilis were detected in pregnant women, with a mean of 3,789 cases/year. The regression model revealed an increase in the detection rate (APC: 19.5%, 95% CI: 17.7-21.4, P < 0.001) from 2.3 to 7.5 pregnant women per 1,000 LB in 2015 (Figure 1), with a ratio of 3.26. The family health team coverage had an increasing trend from 2012 onward (APC: 3.3%, 95% CI: 0.6-6.1, P < 0.001). A strong correlation was observed between the detection rate of syphilis and FHT coverage (0.6672; P < 0.05). Regarding sociodemographic and clinical characteristics, a higher proportion of infection was observed in women in the 20-to-29 age group (15,709 [51.8%]), those of brown ethnicity (19,641 [64.7%]), and those with low educational level (14,872 [49.2%] with primary school as the highest level of educational attainment), and 12,351 (40.7%) syphilis cases were diagnosed during the third trimester of gestation. Among these cases, 11,289 (37.2%) were primary syphilis (Table 1).

**FIGURE 1:**
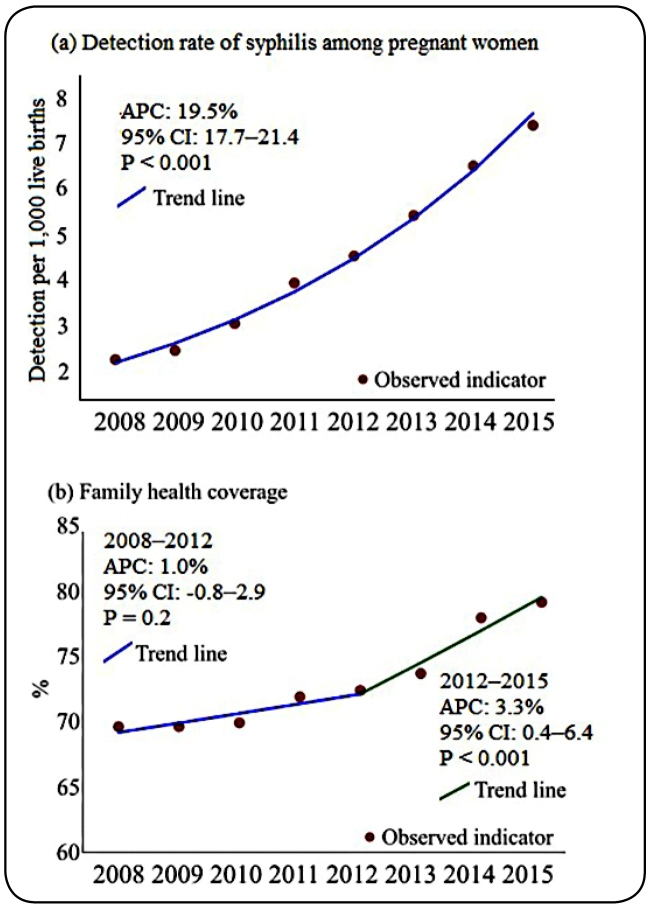
Trend of syphilis detection rate among pregnant women. Northeast Brazil, 2008-2015. Legend: APC- Annual Percent Change; 95% CI- 95% of Confidence Interval.

**TABLE 1:** Sociodemographic characteristics of pregnant women with syphilis. Northeast Brazil, 2008-2015.

Variables		n (%)
Age group	10-14 years	515 (1.7)
	15-19 years	7,205 (23.8)
	20-29 years	15,709 (51.8)
	30-39 years	6,219 (20.5)
	≥40 years	666 (2.2)
Race/color	White	3,511 (11.6)
	Black	3,812 (12.6)
	Caucasian	311 (1.0)
	Brown	19,641 (64.7)
	Indigene	140 (0.5)
	Unknown	2,899 (9.6)
Educational level	Illiterate	721 (2.4)
	Did not complete 1st-4th grade	3,595 (11.9)
	Completed 4th grade	1,904 (6.3)
	Did not complete 5th-8th grade	6,570 (21.7)
	Completed 8th grade	2,082 (6.9)
	Did not complete high school education	2,686 (8.9)
	Completed high school education	2,999 (9.9)
	Did not complete higher school	164 (0.5)
	Completed higher school	126 (0.4)
	Not applicable	9 (<0.0)
	Ignored	9,458 (31.1)
Gestational age	1st trimester	5,201 (17.2)
	2nd trimester	10,262 (33.9)
	3rd trimester	12,351 (40.7)
	Ignored	2,500 (8.2)
Clinical classification	Primary syphilis	11,289 (37.2)
	Secondary syphilis	2,136 (7.1)
	Tertiary syphilis	2,994 (9.9)
	Latent syphilis	4,439 (14.6)
	Ignored	9,456 (31.2)

The regression model showed an increasing trend in the proportion of syphilis in pregnant women aged 15-19 years (APC: 5.3%, 95% CI: 1.9-8.8, P < 0.001), those of brown ethnicity (APC: 1.5%, 95% CI: 0.8-2.2, P < 0.001), and those with higher education level, with emphasis on incomplete degree (APC: −5.8%, 95% CI: −7.6-−4.0, P < 0.001). Furthermore, there were increasing trends in the proportion of cases diagnosed during the first trimester of pregnancy (APC: 5.4%, 95% CI: 2.3-8.5, P < 0.001) and cases of tertiary syphilis (APC: 10.3%, 95% CI: 5.5-15.4, P < 0.001) (Table 2). Conversely, the proportion of cases diagnosed during the second trimester (APC: −2.6, 95% CI: −4.6-−0.6, P < 0.001) and cases of secondary syphilis (APC: −6.3, 95% CI: −9.7-−2.8, P < 0.001) significantly decreased.

**TABLE 2:** Syphilis trend among pregnant women according to sociodemographic and clinical characteristics. Northeast Brazil, 2008-2015.

Variables		Period	APC (95% CI)	Trend
Age group	10-14 years	2008-2015	3.9 (−1.6; 9.7)	Stable
	15-19 years	2008-2012	10.3 (3.8; 17.2)	Increasing Stable
		2012-2015	−1.0 (−10.0; 8.9)	
		2008-2015	5,3 (1,9; 8,8)	Increasing
	20-29 years	2008-2011	−3.8 (−7.1; −0.4)	Decreasing Stable
		2011-2015	0.2 (−2; 2.4)	
		2008-2015	−1,6 (-2.7; −0.4)	Decreasing
	30-39 years	2008-2015	−1.4 (-2.4; −0.5)	Decreasing
	≥40 years	2008-2015	−4.8 (−8.1-−1.3)	Decreasing
Race/color	White	2008-2015	−5.8 (−7.6; −4)	Decreasing
	Black		−0.1 (−2.8; 2.8)	Stable
	Caucasian		4.1 (−5.2; 14.3)	Stationary
	Brown		1.5 (0.8; 2.2)	Increasing
	Indigene		−1.3 (−9.2; 7.4)	Stable
	Ignored		−2.1 (−7.5; 3.6)	Stable
Educational level	Illiterate	2008-2015	−12.6 (−14.9; −10.4)	Decreasing
	Did not complete 1st-4th grade		−8.2 (−10.2; −6.1)	Decreasing
	Completed 4th grade		−8.1 (−10.5; −5.5)	Decreasing
	Did not complete 5th-8th grade		0.7 (−0.9; 2.3)	Stable
	Completed 8th grade		−0.1 (−3.6; 3.6)	Stable
	Did not complete high school		8.1 (5.4; 10.8)	Increasing
	Completed high school		10.4 (7.7; 13.2)	Increasing
	Did not complete higher school		14 (4.8; 24.0)	Increasing
	Completed higher school		5.5 (−1.6; 13.1)	Stable
	Not applicable		-	-
	Ignored		0.7 (−2.0; 3.5)	Stable
Gestational age	1st trimester	2008-2015	5.4 (2.3; 8.5)	Increasing
	2nd trimester		1.4 (−0.1; 2.9)	Stable
	3rd trimester		−2.6 (−4.6; −0.6)	Decreasing
	Ignored		−3.4 (−7.7; 1.0)	Stable
Clinical classification	Primary syphilis	2008-2015	−1.7 (−4.0; 0.6)	Stable
	Secondary syphilis		−6.3 (−9.7; −2.8)	Decreasing
	Tertiary syphilis		10.3 (5.5; 15.4)	Increasing
	Latent syphilis		4.7 (−6.7; 17.6)	Stable
	Ignored		−0.7 (−2.9; 1.5)	Stable

In recent years, the increase in the number of syphilis cases among pregnant women has been a cause of concern among policymakers worldwide^1^ ^,^ ^4^. In Brazil, an increase in the number of cases in multiple states in every region has been reported. However, differences were observed between regions^2^ ^,^ ^5^, with almost a quarter of cases reported in the Northeast Region between 2008 and 2015. In this study, we analyzed the temporal trend of syphilis indicators among pregnant women in Northeast Brazil from 2008 to 2015, and results showed a linear increase in the trend in detection rate, which was strongly correlated to the increase in family health coverage. These results indicate a few implications: a) the expansion of the basic care network has provided pregnant women greater access to the diagnosis of syphilis and b) there is a maintenance of the disease transmission chain, which may be correlated to social vulnerability and unsuccessful diagnosis and treatment even though pregnant women have increased access to diagnosis.

Increased FHS coverage and the development of strategies focusing on maternal and child health have been considered as factors associated with the higher detection rate of such infection among pregnant women^9^ ^-^ ^11^, as observed in our investigation. A study carried out in the state of Goiás in 2014 has shown that the municipalities with the highest family health coverage had the highest incidence rates of syphilis among pregnant women. In the same study, the incidence was 3.5/1,000 LB in municipalities with less than 25% coverage and 5.1/1000 LB in those with more than 75% coverage^8^.

In Northeast Brazil between 2008 and 2015, the proportion of individuals living in households enrolled in a family health unit increased from 67.7% to 75.8%, representing the highest coverage among the Brazilian regions^11^. This increase had a direct impact on greater access to prenatal care. According to the data from the Born in Brazil Survey conducted between 2011 and 2012, the prenatal coverage reached 98.7%, and 73.1% of pregnant women had at least six prenatal appointments^9^. These improvements in coverage and access may explain the possible increase in the proportion of cases detected during the first trimester of gestation, which represents an important advancement in the integral care offered to the maternal and child population.

Conversely, a lack of laboratory tests and/or delayed delivery, pharmaceutical care deficiencies in guaranteeing penicillin treatment, difficulties in maintaining adherence to complete treatment of pregnant woman and her partner, and difficulties in managing the disease due to health problems among health care professionals are some of the main issues observed in the country. These problems are even more severe in poorer states, which is the case in the Northeast Region^9^ ^,^ ^10^. In addition to the maintenance of an chain active, this context implies late diagnosis, as evidenced by the increasing trend in the proportion of tertiary syphilis observed in this study.

The epidemiological profile of pregnant women with syphilis infection contributes to the elucidation of the complexity of syphilis transmission and reveals important nuances of the health-disease process. Among these, the characteristics observed for these cases stand out: young women, between 20 and 29 years of age, of brown ethnicity, with low schooling. In addition, this pattern is in line with other investigations. In the state of Ceará, 56.6% of pregnant women diagnosed from 2008 to 2010 were aged between 20 and 29 years. Moreover, approximately 85.1% were of non-white ethnicity, and 65.1% only had primary education^12^. This profile may be associated with unwanted pregnancy, lack of support from family and partner, and social vulnerability, thereby maintaining the chain of disease transmission^13^.

Although the highest proportion of pregnant women were aged 20-29 years, a statistically significant increasing trend was only observed in the 15-to-19 age group. This finding should be a cause of concern as it indicates a change in the profile of pregnant women with the infection. Factors, such as early onset of sexual activity and inconsistent use of condom, are important social determinants of unwanted gestation and incidence of STI among adolescents^13^. 

An increase in the proportion of syphilis cases among pregnant women of brown ethnicity and a decrease among white pregnant women may reinforce the influence of social context on the dynamics of syphilis transmission among pregnant women. A national study of 36,713 parturient women who were admitted in public or philanthropic maternity wards between 2010 and 2011 has shown that pregnant women of non-white ethnicity had a two-fold increased risk of syphilis infection compared with white pregnant women^14^. 

In the Northeast Region, there was a high proportion of pregnant women with syphilis who have low educational level (< 8 years of education), which is in accordance with the result of the current literature^12^ ^,^ ^14^. A national research has shown that the risk of infection among illiterate pregnant women is 3.94 times higher than that among women with a higher educational level^14^.

The decreasing trend in the proportion of pregnant women with low educational level, accompanied by an increase in the proportion of pregnant women with a higher educational level, may reflect access to health services. An analysis of almost 48 million births registered in Brazil between 2000 and 2015 has revealed that the number of prenatal consultations performed was higher in pregnant women with a higher level of education^15^. Knowledge about the rights and importance of prenatal consultations as well as better living conditions experienced by this part of the population may justify this finding^15^. Associations between ethnicity, education, and income as well as access to services may have an important impact on the maintenance of syphilis transmission.

Regardless of methodological care, this study had some limitations. As data were obtained from different databases, they might have been influenced by the quality of local surveillance services. Furthermore, the number of cases may have been underreported, particularly in small municipalities with low FHS coverage or with difficulties in carrying out laboratory tests. However, although the study had limitations, our findings are valuable, considering that they refer to more than 30,000 cases of syphilis among pregnant women during an 8-year study period.

In summary, an increasing trend of syphilis cases among pregnant women, particularly in young women of brown ethnicity with low educational level, in the Northeast Region was observed. This increasing trend was strongly correlated to greater FHS coverage. Based on this result, there is an urgent need to develop systematic and integrated actions that allow for timely diagnosis and treatment of pregnant women and their partners as well as actions that reduce the social inequalities involved in the maintenance of disease transmission dynamics in the region.
